# The representational bottleneck in rehabilitation AI: from human-cognitive proxies to pathway-based clinical representations

**DOI:** 10.3389/fdgth.2026.1917999

**Published:** 2026-08-25

**Authors:** Eun Joo Yang, Seung Hyun Chung

**Affiliations:** 1 Department of Rehabilitation Medicine, Seoul National University Bundang Hospital, Seongnam, Republic of Korea; 2 Department of Physical Medicine and Rehabilitation, National Cancer Center, Goyang, Republic of Korea

**Keywords:** clinical reality translation-construction, digital biomarkers, digital phenotyping, human-cognitive proxy, pathway-based clinical representation, rehabilitation AI, rehabilitation medicine, representational bottleneck

## Abstract

Artificial intelligence (AI) in healthcare is often framed as a problem of model performance. This view overlooks a prior representational problem: many clinical data are human-cognitive proxies built for communication, documentation, classification, or administration rather than high-fidelity computational access to clinical phenomena. In rehabilitation medicine, movement quality, compensatory strategy, sensorimotor integration, fatigue-related deterioration, and postural instability are frequently compressed into proxy measures such as TUG, BBS, FMA, ECOG performance status, diagnosis codes, or clinical notes. We propose Clinical Reality Translation-Construction (CRTC), a representational design framework that specifies how clinically meaningful phenomena can be constructed as AI-readable entities before model development. CRTC argues that the bottleneck in rehabilitation AI is often representational before it is algorithmic. Its central task is not merely to automate interpretation of existing human-readable proxies, but to construct pathway-based clinical representations: AI-readable entities that model how clinical problems emerge through bodily impairment, temporal adaptation, environmental coupling, habitual compensation, symptom perception, institutional classification, and lived disability. Drawing on digital phenotyping, digital biomarkers, ambient AI documentation, imaging AI, digital twins, wearable monitoring, and rehabilitation kinematics, this Perspective outlines CRTC and criteria for evaluating whether richer representations improve prediction, explanation, actionability, and equitable implementation. CRTC therefore shifts the primary design question from how AI should learn to what should become available for learning.

## 1 Introduction: the overlooked representational layer

Artificial intelligence (AI) has become central to digital health, with applications in imaging diagnosis, risk prediction, ambient documentation, remote monitoring, and multimodal oncology. Much discussion appropriately focuses on accuracy, validation, workflow integration, safety, and bias. These issues, however, presuppose that the data made available to AI are adequate representations of the clinical phenomena being modeled.

This assumption is fragile. Electronic health records (EHRs), diagnostic codes, clinical notes, performance scores, and rehabilitation scales are not neutral mirrors of clinical reality. They are products of measurement systems, professional classifications, documentation workflows, institutional incentives, and human judgment. Reviews of hospital AI implementation emphasize that moving from static retrospective datasets to live learning health systems remains difficult, with workflow and infrastructure shaping whether AI improves care (1). Bias reviews similarly show that EHR data contain distortions arising from missingness, coding practices, clinician bias, access disparities, device bias, labeling practices, and downstream algorithmic feedback (2, 3).

Rehabilitation medicine makes this representational problem particularly visible. A patient may experience instability during turning, fatigue-related deterioration across repeated tasks, altered sensory reliance, compensatory trunk recruitment, or fear-driven activity restriction. Yet what enters the record is often a Timed Up and Go (TUG) time, Berg Balance Scale (BBS) score, Fugl-Meyer Assessment (FMA) score, ECOG or Karnofsky performance status, grip strength value, or note such as “gait disturbance.” These are indispensable clinical tools, but they are also human-cognitive proxies: useful, communicable, and institutionally tractable representations that compress embodied, dynamic, and temporally evolving phenomena.

CRTC does not assume access to unmediated clinical reality. Rather, it argues that representations differ in their ability to preserve the pathways through which clinical problems arise and become consequential. Here, “pathway-based” does not refer to standardized care pathways or clinical protocols, but to representations that preserve how clinical problems emerge through bodily impairment, temporal change, behavioral adaptation, environmental context, clinical interpretation, and documentation. The central claim is that the next advance in rehabilitation AI requires moving upstream from automated interpretation of existing proxies toward the construction of AI-readable clinical entities grounded in pathway-based clinical representation.

## 2 Human-cognitive proxies in rehabilitation medicine

A human-cognitive proxy is a clinical representation designed primarily for human perception, communication, classification, documentation, or administration rather than for high-fidelity computational access to the underlying phenomenon. Human-cognitive proxies in rehabilitation can be broadly grouped by purpose: administrative proxies such as diagnosis codes and reimbursement classifications compress clinical problems for institutional coordination; clinical rating proxies such as the TUG, BBS, FMA-UE, MBI, and ECOG compress observations into scores for communication and documentation; and narrative proxies such as clinical notes and imaging reports compress encounter content into interpretable records. The problem is not their existence but their repurposing: tools designed for human communication and institutional coordination are often reused as if they were high-fidelity computational access points to the underlying clinical phenomenon. Whether AI systems learn from explicit proxy labels or derive latent representations from large unstructured datasets, they can only access the clinical phenomena that data collection infrastructures have made available. When those infrastructures consist primarily of proxies, the representational structure those proxies encode—not the structure of the clinical phenomena themselves—shapes what any downstream model can learn.

Stroke rehabilitation makes this visible across core instruments. The Modified Barthel Index (MBI) and broader ADL/IADL assessments capture whether a task is performed, but not how: the compensatory strategies used, cognitive resources recruited, environmental modifications required, or fatigue-related variability across the day. Brunnstrom stages classify synergy patterns, and the Fugl-Meyer Assessment for Upper Extremity (FMA-UE) measures volitional movement, but both compress information needed to distinguish motor restoration from compensatory strategy. Machine learning models predicting upper-limb recovery from FMA-UE scores, imaging, and neurophysiology can achieve meaningful discrimination, yet still do not access movement quality, trunk compensation, or the trajectory of motor reorganization (4). Inertial sensor studies during the Box and Block Test show that 84%–88% of post-stroke individuals use substantial wrist, shoulder, or trunk compensation that conventional scores cannot detect (5). Thus, two patients may obtain the same FMA-UE score through different mechanisms—distal recovery in one case and proximal compensation in another—while proxy-based AI treats them as equivalent. Cognitive impairment, neglect, apraxia, mood, environmental barriers, and participation context add further pathways that conventional datasets often record as separate domain scores rather than as connected mechanisms of recovery or disability.

A parallel gap appears in cancer survivorship rehabilitation. A multicenter randomized trial of a sensorimotor, movement-quality-focused intervention improved functional mobility and postural stability in breast cancer survivors without corresponding changes in grip strength, six-minute walk distance, or self-reported disability (6), suggesting that conventional proxies and rehabilitation-relevant change may measure different constructs. Free-living upper-limb sensor data further show that automated activity metrics may overestimate functional arm use, requiring pathway-level interpretation rather than aggregate counts (7).

Breast cancer-related lymphedema (BCRL) illustrates that the representational bottleneck is not unique to AI; it often begins in conventional clinical classification itself. Existing staging captures clinically important endpoints such as visible swelling, reversibility, and tissue remodeling (8), while bioimpedance spectroscopy, indocyanine green lymphography, and ultrasound can characterize fluid burden, lymphatic transport, and tissue composition (9–11). However, without a conceptual representation that links these dimensions to symptom burden and functional limitation, they remain separate clinical indicators rather than a connected disease pathway. AI trained on such data would therefore amplify an existing medical representational problem: it would learn fragmented markers of BCRL status rather than the pathway through which lymphatic injury becomes fluid accumulation, tissue remodeling, symptoms, and lived functional consequence.

## 3 Current medical AI as second-order translation

**Figure 1:**
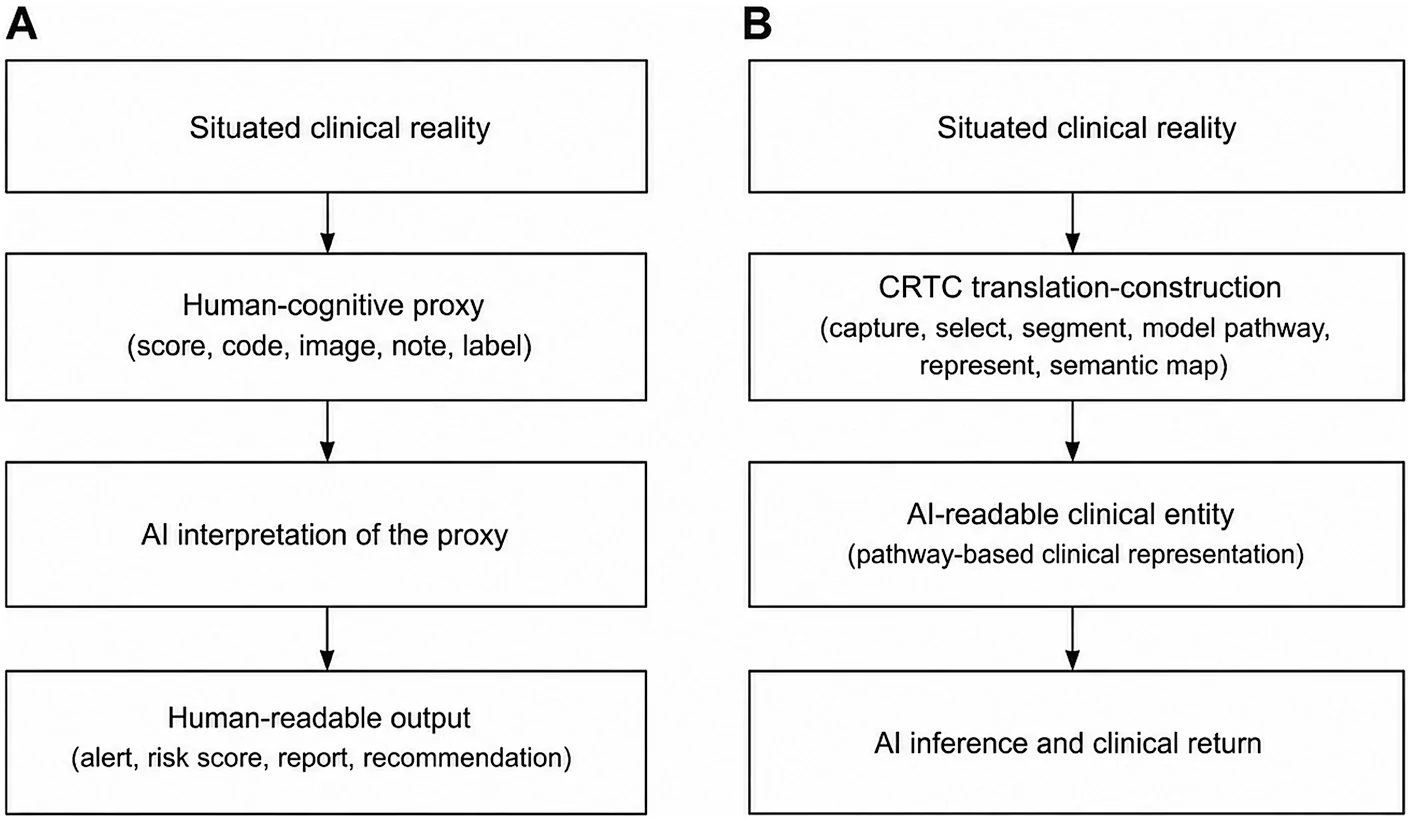
Current medical AI as second-order translation vs. Clinical Reality Translation-Construction (CRTC). **(A)** In conventional medical AI, situated clinical reality is first translated into human-cognitive proxies (e.g., scores, codes, images, notes, or labels). AI systems then learn from these proxies and generate human-readable outputs such as risk scores, alerts, reports, or recommendations. **(B)** In CRTC, situated clinical reality is captured, segmented, modeled as a clinical pathway, and translated into AI-readable clinical entities before AI inference. These pathway-based representations are intended to preserve clinically meaningful structures that may be compressed or lost in conventional proxy-based approaches.

Much of current medical AI can be understood as second-order translation. First, clinical reality is translated into a human-oriented proxy: image, note, code, score, label, or report. Second, AI interprets that proxy and returns another human-readable output such as a risk score, alert, label, heatmap, generated note, or recommendation (Figure 1).

Medical imaging AI illustrates this pattern. Diagnostic images encode physiological and pathophysiological information, and AI can extract subvisual or nonvisual patterns, but such systems still operate within image acquisition practices, labeling conventions, demographic and technical biases, and learned correlations that may lack causal grounding (12). Ambient AI scribes provide another example: their primary output remains a documentation proxy inserted into the EHR (13). EHR-based representation learning, including clinical code embeddings and knowledge graphs, can bridge gaps between administrative vocabularies and clinical reasoning (14), while ontology-aware design patterns can address documentation distortion, terminology drift, and reification loops (15). These efforts are important, but they mainly improve representations of already documented entities. CRTC asks an upstream question: how do situated clinical phenomena become represented before they enter code, score, image, or note infrastructures?

## 4 Clinical reality translation-construction

CRTC is a representational design framework. It is not a sensor technology, biomarker category, modeling architecture, or digital twin specification. It is a pre-modeling representational framework that specifies what clinical phenomenon should be made computable, at what scale, and through which pathway structure before data collection, feature engineering, or model training. Rather than replacing existing digital health methodologies, it provides upstream design criteria for what those methods should represent. The same sensor stream, EHR database, or machine learning architecture can therefore produce different clinical entities depending on the prior representational target.

### 4.1 Clinical entity layer: what is made available for computation?

The first layer asks which clinical objects, categories, states, and relationships a data infrastructure makes available for computation. EHRs, scales, and codes make diagnoses, procedures, medications, and scores visible while leaving hesitation, compensatory movement, sensory dependence, activity avoidance, or instability under fatigue weakly visible. In rehabilitation, potential AI-readable entities include a compensation-dominant upper-limb phenotype, fatigue-linked motor degradation phenotype, sensory-dependence profile, transition-instability phenotype, or symptom-function coupling phenotype.

### 4.2 Knowledge and measurement layer: how does AI know the phenomenon?

AI does not learn clinical reality directly. It learns the representations made available by data infrastructure. Before asking whether a model is accurate, clinicians and data scientists should ask whether the input representation captures the phenomenon that matters. A model predicting falls from diagnosis codes, prior falls, and BBS scores may be useful, but it differs from a model that captures sensory conflict, delayed correction, task-transition instability, environmental hazards, and fear-driven avoidance. The BBS, for example, compresses sway dynamics, correction latency, sensory reweighting, and transition instability into a 14-item ordinal total that categorizes fall risk—a clinically practical representation that does not preserve the pathway through which balance fails (16).

### 4.3 Pathway layer: how is the clinical problem produced?

Medicine rarely asks only “What appears?” It asks “How did this problem come to be?” Although similar questions have been explored in broader philosophical accounts of how phenomena are constituted (17, 18), CRTC approaches them pragmatically as a representation-design problem for AI-enabled rehabilitation.

Two examples illustrate this in rehabilitation. In stroke, corticospinal tract disruption may generate pathological synergy; reaching attempts then recruit trunk rotation or shoulder elevation, and repeated use may consolidate compensation. Brunnstrom stage, MBI, or FMA-UE total scores document the current endpoint but not how the patient arrived there or which compensation trajectory is developing. A model trained on such proxy-derived data learns to predict score categories rather than the underlying motor reorganization process, regardless of its architecture (4).

In cancer survivorship, chemotherapy-related sensory impairment may alter somatosensory feedback and postural control, increasing transition instability, visual dependence, fear of falling, activity restriction, deconditioning, and mobility loss before being reduced to labels such as “gait abnormality,” “fall,” or “depression” (19). Symptom-function relationships show a similar layered structure: experienced symptoms may shape somatic sensations, while somatic sensations relate more directly to disability and functioning (20).

### 4.4 Technical-operational layer: how is the representation built?

CRTC can be summarized as a nine-stage design heuristic: (1) capture the clinical situation through video, wearables, apps, audio, EHR, patient-reported outcomes, clinician observation, or environmental data; (2) select the clinically meaningful phenomenon; (3) segment continuous reality into meaningful events; (4) model the pathway through impairment, adaptation, compensation, environment, perception, habit, and institutional classification; (5) represent the pathway as time series, trajectories, graphs, embeddings, state-space variables, or multimodal structures; (6) map features to clinical meanings; (7) construct computable phenotypes; (8) perform AI inference; and (9) return outputs to practice.

These steps are not individually novel technical operations. Their contribution is their ordering around a specific representational question: is the AI system merely interpreting existing proxies, or is it constructing clinically meaningful entities before modeling begins? The nine stages correspond to the CRTC pipeline shown in Figure 1B. Stage 7 deliberately borrows the term computable phenotype, but CRTC uses it more broadly than EHR-based case definitions—forming clinically meaningful AI-readable entities from situated functional, behavioral, sensorimotor, and symptom-related phenomena. A brief walk-through illustrates the approach. For post-stroke upper-limb dysfunction, the conventional proxy is an FMA-UE total score. A CRTC-informed representation segments the reaching attempt into initiation, transport, grasp, and release phases, extracting trunk displacement, shoulder elevation, wrist stabilization, inter-joint timing, and fatigue-related degradation per trial. These map to a compensation-dominant vs. restoration-dominant reaching phenotype—an AI inference target that predicts real-world arm use and therapy response in ways an FMA-UE score cannot differentiate. Extended walk-throughs are in Supplementary Boxes 1-2. Supplementary Box 1 provides a complete nine-stage implementation example in post-stroke upper-limb rehabilitation. Beginning with video and inertial-sensor capture during reaching, the example segments task phases, quantifies trunk and upper-limb kinematics, maps these features to compensatory motor strategies, constructs a compensation-dominant computable phenotype, and links that phenotype to AI-supported inference and rehabilitation decision-making. Thus, the nine stages are intended not as sequential technical requirements, but as an end-to-end representational design workflow connecting clinical observation to computational inference and clinical return.

### 4.5 Criteria for pathway-based clinical representations

A representation should not be considered pathway-based simply because it is digital, high-dimensional, sensor-derived, or predictive. We propose six criteria (Table 1).

**Table 1:** Criteria for pathway-based clinical representations.

Criterion	Meaning
Mechanistic relevance	It relates to a plausible mechanism generating the clinical phenomenon.
Temporal trajectory integrity	It preserves not only that change occurred but the structure of the trajectory: the sequence, rate, direction, and inter-state transitions through which the clinical configuration evolved over time.
Contextual responsiveness	It changes meaningfully across task, environment, fatigue, symptom, or social context.
Clinical interpretability	It can be semantically mapped to clinically meaningful constructs.
Additive explanatory or predictive value	It explains or predicts outcomes beyond existing proxies.
Scale-explicit representation	It specifies the temporal, spatial, and sampling scale at which the phenomenon is captured, and documents what is unresolved or lost at adjacent scales.

Digital biomarker validation criteria—validity, generalizability, responsiveness, and outcome associations (21, 22)—provide a partial precedent, but address whether a feature is predictive rather than which clinical phenomenon it makes computable. CRTC extends this logic upstream to representation design.

## 5 Relationship to existing digital health frameworks

CRTC occupies a distinct upstream position relative to digital phenotyping, digital biomarkers, multimodal AI, digital twins, and patient representation learning. These frameworks typically begin with data streams, validated digital features, embeddings, simulation targets, or category membership. CRTC begins earlier, with a clinical-representational question: what pathway-structured clinical entity should exist for those methods to model? Digital phenotyping asks what continuous data characterize behavior or physiology; digital biomarkers ask what digital feature is clinically valid; digital twins ask how patient state can be simulated; computable phenotypes ask which patients fit a clinical category. CRTC provides the design criteria that determine what these methods are asked to represent (Table 2). The novelty of CRTC therefore lies not in introducing another computational method, but in formalizing the upstream design step through which a clinically meaningful phenomenon is selected, pathway-structured, and made computable before digital phenotyping, biomarker validation, representation learning, multimodal integration, or simulation begins.

**Table 2:** Relationship between CRTC and adjacent digital health frameworks.

Framework	Starting point	Primary aim/output	Relationship to CRTC
Digital phenotyping	Sensor/digital streams	Behavioral or physiological patterns	Characterizes digitally observable states; CRTC specifies which clinically meaningful phenomenon should first be represented.
Digital biomarkers	Digital features	Validated clinical indicator	Validates measurable features; CRTC specifies the clinical entity and pathway to which the feature belongs.
Computable phenotypes	Recorded clinical/EHR variables	Computable identification of clinical states or cohorts	Operationalizes predefined clinical categories; CRTC addresses how clinically meaningful entities are constructed before categorization.
Patient representation learning	Recorded patient data	Learned embeddings or latent representations	Learns representations from available data; CRTC asks what clinical entities should be made available before representation learning.
Digital twins	Patient state variables	Dynamic simulation or prediction	Simulates represented patient states; CRTC specifies how clinically meaningful state variables are constructed.
Multimodal AI	Multiple data modalities	Integrated prediction or inference	Integrates available modalities; CRTC specifies what clinically meaningful entities those modalities are intended to represent.
CRTC	Clinical phenomenon and its generating pathway	Pathway-based AI-readable clinical entity	Upstream representational design before model development.

Digital phenotyping studies support CRTC but do not resolve when a collected sensor stream becomes a meaningful representation rather than another proxy (23, 24). Digital twins integrate multiscale data and simulate trajectories, but a twin built on proxy-level inputs—ECOG scores, ICD codes, aggregate step counts—will simulate a proxy-level patient, not the functional trajectory of situated clinical reality (25, 26). Patient representation learning and multimodal AI face the same upstream problem: learned embeddings or integrated modalities remain constrained by the clinical entities made available for modeling (27–29).

CRTC should not be understood as a competing machine-learning methodology, digital biomarker framework, digital twin architecture, or representation-learning approach. Rather, it operates at a prior representational layer by specifying what clinical phenomenon should become an AI-readable entity before those methods are applied. Consequently, CRTC does not replace existing digital health methodologies; it provides design criteria for determining what they are asked to represent.

## 6 Discussion: research agenda, design principles, governance, and limitations

### 6.1 Empirical validation

The central empirical test of CRTC is whether pathway-based representations add explanatory, predictive, or actionable value beyond conventional proxies. Evaluation should include discrimination, calibration, temporal generalizability, subgroup robustness, interpretability, and decision consequences. As illustrated by the stroke example, validation should test whether models distinguish restoration from compensation rather than only predict global scores (5). A practical proof-of-concept study could proceed in three phases: first, derive a transition-instability phenotype from wearable or video features and compare it with TUG or BBS scores; second, test whether the phenotype predicts falls, mobility decline, or activity restriction beyond conventional scores; and third, evaluate whether phenotype-informed intervention selection improves decision consequences or rehabilitation response. In fall-risk assessment, conventional scores may obscure the behavioral and environmental pathways that drive instability (30). Remote monitoring and aging-in-place technologies similarly require a distinction between clinically meaningful representations and accumulated sensor streams (31). Incremental value should be evaluated explicitly against a conventional-proxy baseline. Depending on the clinical task, measurable evidence may include improvement in discrimination (e.g., change in AUROC), calibration, explained variance, prediction error, or longitudinal responsiveness after adding the pathway-based representation to a model containing conventional scores alone. Beyond predictive performance, discordance analysis can test whether patients with similar conventional scores are separated into clinically meaningful phenotypes with different compensation, instability, fatigue-related deterioration, or subsequent outcomes. Decision-curve analysis or changes in clinically relevant risk classification may further assess whether the additional representation alters clinical decisions rather than merely improving statistical fit.

Three illustrative validation pathways may help operationalize CRTC.

First, a transition-instability phenotype could be derived from wearable or video-based features during gait initiation, turning, and task transitions. Validation would test whether the phenotype predicts falls, mobility decline, or activity restriction beyond conventional proxies such as TUG or BBS scores.

Second, a compensation-dominant reaching phenotype could be constructed from upper-limb kinematics, including trunk displacement, shoulder elevation, inter-joint coordination, and fatigue-related degradation. Validation would test whether the phenotype distinguishes motor restoration from compensation and predicts real-world arm use or rehabilitation responsiveness beyond FMA-UE scores.

Third, a symptom-function coupling phenotype could be derived from multimodal symptom reports, sensorimotor performance measures, and functional outcomes in cancer survivorship. For example, movement-quality-focused interventions may improve mobility and postural control despite minimal changes in conventional strength or disability measures. Validation would test whether pathway-based representations better capture clinically meaningful change than existing proxy measures alone.

These examples illustrate how CRTC can generate empirically testable representations across different rehabilitation domains, providing a framework for evaluating whether pathway-based entities offer explanatory, predictive, or actionable value beyond conventional clinical proxies.

### 6.2 Design principles for clinical teams

For clinical teams, the practical question should shift from “How can AI automate what I already score?” to “What clinically important process does my current score fail to represent?” This reframing changes AI design from score automation to clinical entity construction. Three design principles follow. First, representation should begin with a clinical process that existing scores fail to capture, rather than with an available sensor stream. Second, digital features should be linked to a plausible pathway of functional loss, compensation, adaptation, or recovery. Third, validation should test whether the new representation adds explanatory or predictive value beyond conventional scores. Rehabilitation-specific examples of proxy measures and pathway-based representations are provided in Supplementary Table S1 and Figure S1.

### 6.3 Governance and implementation

CRTC also has governance implications. Richer representations increase privacy risk because kinematic profiles, ambient audio, location traces, continuous activity data, and symptom trajectories can reveal information beyond the intended scope of care. Bias can arise across data collection, labeling, model development, evaluation, deployment, publication, and user interaction (3). A further risk is reification: a computable entity such as a “compensation phenotype,” “instability score,” or “sensory-dependence profile” may be treated as a natural fact rather than as a representation requiring validation. Periodic auditing, recalibration, and explicit versioning are therefore needed to keep pathway-based representations provisional. Although developed in rehabilitation medicine, the representational problem described here is unlikely to be unique to rehabilitation. Similar challenges may arise in chronic pain, psychiatry, frailty, long COVID, and other conditions where dynamic adaptation, contextual dependency, and lived functional consequences are weakly preserved in conventional clinical data infrastructures.

Routine implementation also requires attention to infrastructure, standardization, and interoperability. Video-, wearable-, and multimodal representations may require device calibration, secure storage, computational processing, quality-control procedures, and interfaces with EHR and clinical information systems. Cross-site deployment further requires standardized acquisition protocols, feature definitions, metadata, and semantic mappings so that a pathway-based entity retains comparable clinical meaning across devices and institutions. These requirements create costs in equipment, personnel training, processing time, governance, and maintenance that must be weighed against the incremental clinical value of the representation. Importantly, CRTC does not imply maximal multimodal data collection. Representational complexity should be proportional to the clinical question: the least burdensome set of measurements that adequately preserves the clinically relevant pathway may be preferable to indiscriminate high-dimensional capture.

### 6.4 Limitations

Several limitations should be explicit. CRTC is a Perspective framework, not an empirical finding, and does not claim that CRTC-derived representations already outperform conventional proxies. Its contribution is to define the representational problem, specify operational criteria, and outline testable hypotheses. The distinction between proxy and pathway-based representation is one of degree, not kind, and can be evaluated using the criteria in Table 1. The pragmatic burden is also substantial: richer representations require infrastructures for high-dimensional temporal data. These requirements caution against equating technically improved AI models with clinical efficiency, because even accurate systems may increase human work when implementation, monitoring, maintenance, and workflow integration are considered (32). Modern representation-learning approaches, including self-supervised and foundation-model approaches, may derive latent representations without explicit proxy supervision (28, 29), potentially capturing structure that labeled approaches miss. However, these methods can only access phenomena that data collection infrastructures have recorded; if rehabilitation data infrastructures consist primarily of scores, codes, and notes, even such models will learn within the representational boundaries those infrastructures define. Without a specified representational target, high-dimensional data collection may simply produce richer proxies. Additional limitations deserve consideration. First, pathway construction itself may be influenced by investigator assumptions, meaning that different teams may construct different representations of the same clinical phenomenon. Second, richer representations are not necessarily superior representations; increasing representational complexity may reduce feasibility, scalability, or clinical usability. Third, some pathway-based representations may remain difficult to operationalize within routine clinical workflows despite their theoretical value.

Importantly, CRTC is not intended as an unfalsifiable conceptual position. Its central claim is that pathway-based clinical representations should provide explanatory, predictive, or actionable value beyond conventional proxy measures. If such benefits are not observed across empirical evaluations, the practical justification for CRTC would be substantially weakened. The framework therefore advances a testable hypothesis rather than a purely theoretical assertion.

## 7 Conclusion

The next stage of rehabilitation AI should not be defined only by more accurate models, larger datasets, or more efficient automation of existing documentation. It should also be defined by better representations of clinical reality. Rehabilitation medicine is particularly important for this shift because its core phenomena—movement quality, compensation, postural control, fatigue, adaptation, participation, and disability—are weakly preserved in conventional data infrastructures.

Clinical Reality Translation-Construction offers a framework for this missing representational layer. It asks how clinical phenomena are selected, segmented, modeled, represented, semantically mapped, and returned to practice as AI-readable clinical entities. The future of AI-enabled rehabilitation will depend not only on better algorithms, but on the construction of more clinically meaningful representations for those algorithms to learn from. The central challenge for the next generation of clinical AI may not be how machines learn, but what clinical reality is made available for learning in the first place.

## Data Availability

The original contributions presented in the study are included in the article/Supplementary Material, further inquiries can be directed to the corresponding author.

## References

[1] Kamel Rahimi A Pienaar O Ghadimi M Canfell OJ Pole JD Shrapnel S. Implementing AI in hospitals to achieve a learning health system: systematic review of current enablers and barriers. J Med Internet Res. (2024) 26:e49655. 10.2196/49655 39094106 PMC11329852

[2] Perets O Stagno E Ben Yehuda E McNichol M Celi LA Rappoport N. Inherent bias in electronic health records: a scoping review of sources of bias. ACM Trans Intell Syst Technol. (2025). 10.1145/375792440575765

[3] Cross JL Choma MA Onofrey JA. Bias in medical AI: implications for clinical decision-making. PLOS Digit Health. (2024) 3:e0000651. 10.1371/journal.pdig.0000651 39509461 PMC11542778

[4] Park JM Lee SC Kim YW Yoon SY. Machine learning-based prediction of transition to functional upper limb recovery after intensive inpatient rehabilitation in early subacute stroke. J Clin Med. (2026) 15:3851. 10.3390/jcm15103851 42194811 PMC13207809

[5] Thouant C-L Cocco ES Morone G Manzia CM Infarinato F Romano P. Compensation strategies in post-stroke individuals: insights from upper body kinematics analysis based on inertial sensors. Sensors. (2025) 25:7609. 10.3390/s25247609 41471605 PMC12736794

[6] Yang EJ Kim HS Jeon HR Suh MR Ahn SY Yoon JA. Effects of a sensorimotor-based, movement quality-focused intervention on functional mobility in breast cancer survivors: a multicenter randomized controlled trial. Breast Cancer Res Treat. (2026) 216:37. 10.1007/s10549-026-07952-2 41874709

[7] Vets N De Groef A Verbeelen K Devoogdt N Smeets A Van Assche D. Assessing upper limb function in breast cancer survivors using wearable sensors and machine learning in a free-living environment. Sensors. (2023) 23:6100. 10.3390/s23136100 37447951 PMC10347074

[8] Executive Committee of the International Society of Lymphology. The diagnosis and treatment of peripheral lymphedema: 2020 consensus document of the international society of lymphology. Lymphology. (2020) 53:3–19. 10.2458/lymph.4649 32521126

[9] Kassamani YW Brunelle CL Gillespie TC Bernstein MC Bucci LK Nassif T. Diagnostic criteria for breast cancer-related lymphedema of the upper extremity: the need for universal agreement. Ann Surg Oncol. (2022) 29:989–1002. 10.1245/s10434-021-10645-3 34505218

[10] Sanderson J Tuttle N Box R Reul-Hirche H Laakso E-L. Localised objective characterisation assessment of lymphoedema (LOCAL): using high-frequency ultrasound, bioelectrical impedance spectroscopy and volume to evaluate superficial tissue composition. Diagnostics. (2024) 14:1616. 10.3390/diagnostics14151616 39125492 PMC11311978

[11] Soran A Bengur FB Rodriguez W Chroneos MZ Sezgin E. Early detection of breast cancer-related lymphedema: accuracy of indocyanine green lymphography compared with bioimpedance spectroscopy and subclinical lymphedema symptoms. Lymphat Res Biol. (2023) 21:166–73. 10.1089/lrb.2022.0066 36946918

[12] McLeod GA Stanley EAM Rosenal T Forkert ND. Distinct visual biases affect humans and artificial intelligence in medical imaging diagnoses. NPJ Digit Med. (2026) 9:62. 10.1038/s41746-025-02226-5 PMC12820067 41430423

[13] Ohde JW Thompson A Liu Z Rost LM Overgaard JD Tan JYE. Barriers and opportunities of scaling ambient AI scribes for clinical documentation across diverse healthcare settings. npj Digit Med. (2026) 9:369. 10.1038/s41746-026-02554-0 41866429 PMC13172454

[14] Johnson R Gottlieb U Shaham G Eisen L Waxman J Devons-Sberro S. Embeddings of clinical codes enable knowledge-grounded AI in medicine. NPJ Digit Med. (2026). 10.1038/s41746-026-02664-9 PMC13658233 42277300

[15] Stummer FO. Ontology-aware design patterns for clinical AI systems: translating reification theory into software architecture. arXiv:2604.01661. [Preprint]. (2026). 10.48550/arXiv.2604.01661

[16] Joa KL. Outcome measurement in balance problems: berg balance scale. Ann Rehabil Med. (2024) 48:103–4. 10.5535/arm.240029 38649325 PMC11058368

[17] Husserl E. Analyses Concerning Passive and Active Synthesis: Lectures on Transcendental Logic. Dordrecht: Kluwer Academic Publishers (2001).

[18] Steinbock AJ. Home and Beyond: Generative Phenomenology After Husserl. Evanston, IL: Northwestern University Press (1995).

[19] Namoos A Thomson N Olson C Sheppard V Aboutanos M. Physical and psychological burdens among breast cancer survivors: evaluating post-treatment gait impairment, falls, and depression using real-world data. Eur J Cancer Care. (2025) 2025:5558563. 10.1155/ecc/5558563 PMC12588077 41200408

[20] Yang EJ Lee KS Lim MC Baek JY Han J-Y Yu E-S. Symptom perception and functioning in patients with advanced cancer. PLoS One. (2021) 16:e0245987. 10.1371/journal.pone.0245987 33539372 PMC7861403

[21] Lu JK Wang W Mahadzir MDA Poganik JR Moqri M Herzog C. Digital biomarkers of ageing for monitoring physiological systems in community-dwelling adults. Lancet Healthy Longev. (2025) 6:100725. 10.1016/j.lanhl.2025.100725 40517785

[22] Zhai S Liaw A Shen J Xu Y Svetnik V FitzGerald JJ. A novel machine learning based framework for developing composite digital biomarkers of disease progression. Front Digit Health. (2025) 6:1500811. 10.3389/fdgth.2024.1500811 39867644 PMC11760596

[23] Zhang Y Wang J Zong H Singla RK Ullah A Liu X. The comprehensive clinical benefits of digital phenotyping: from broad adoption to full impact. npj Digit Med. (2025) 8:196. 10.1038/s41746-025-01602-5 40195396 PMC11977243

[24] Jenciute G Kasputyte G Buneviciene I Korobeinikova E Vaitiekus D Inciura A. Digital phenotyping for monitoring and disease trajectory prediction of patients with cancer: protocol for a prospective observational cohort study. JMIR Res Protoc. (2023) 12:e49096. 10.2196/49096 37815850 PMC10599285

[25] Hernandez-Boussard T Macklin P Greenspan EJ Gryshuk AL Stahlberg E Syeda-Mahmood T. Digital twins for predictive oncology will be a paradigm shift for precision cancer care. Nat Med. (2021) 27:2065–6. 10.1038/s41591-021-01558-5 34824458 PMC9097784

[26] Silva A Vale N. Digital twins in personalized medicine: bridging innovation and clinical reality. J Pers Med. (2025) 15:503. 10.3390/jpm15110503 41295204 PMC12653454

[27] Dellamonica D Ruau D Griffiths B Rossi G Li BT Razavi P. The AI revolution: how multimodal intelligence will reshape the oncology ecosystem. npj Artif Intell. (2025) 1:40. 10.1038/s44387-025-00044-4

[28] Moor M Banerjee O Abad ZSH Krumholz HM Leskovec J Topol EJ. Foundation models for generalist medical artificial intelligence. Nature. (2023) 616:259–65. 10.1038/s41586-023-05881-4 37045921

[29] Zheng Y Bensahla A Bjelogrlic M Zaghir J Turbe H Bednarczyk L. A scoping review of self-supervised representation learning for clinical decision making using EHR categorical data. npj Digit Med. (2025) 8:362. 10.1038/s41746-025-01692-1 40517140 PMC12167381

[30] Gonzalez-Castro A Leiros-Rodriguez R Prada-Garcia C Benitez-Andrades JA. The applications of artificial intelligence for assessing fall risk: systematic review. J Med Internet Res. (2024) 26:e54934. 10.2196/54934 38684088 PMC11091813

[31] Sapci AH Sapci HA. Innovative assisted living tools, remote monitoring technologies, artificial intelligence-driven solutions, and robotic systems for aging societies: systematic review. JMIR Aging. (2019) 2:e15429. 10.2196/15429 31782740 PMC6911231

[32] Jongsma KR Sand M Milota M. Why we should not mistake accuracy of medical AI for efficiency. NPJ Digit Med. (2024) 7:57. 10.1038/s41746-024-01047-2 38438477 PMC10912629

