# Supplementary material for: The representational bottleneck in rehabilitation AI: from human-cognitive proxies to pathway-based clinical representations
Source: Front Digit Health. 2026 Aug 25;8:1917999. doi: 10.3389/fdgth.2026.1917999 (PMC13550172; doi:10.3389/fdgth.2026.1917999)
Supplement: Supplementary file 1 [file Datasheet1.docx]

### Supplementary Figure 1. **Conceptual comparison between proxy-based AI and pathway-based clinical representation in rehabilitation. (A)** Conventional rehabilitation AI relies on proxy outcomes and labels that compress complex clinical reality into simplified scores or categories. **(B)** The CRTC framework preserves clinically meaningful pathways linking impairment, adaptation, performance degradation, and disability, enabling digital signals to be translated into AI-readable clinical entities rather than isolated features.

# Supplementary Table 1. Human-cognitive proxies and pathway-based clinical representations in rehabilitation

| Domain | Human-cognitive proxy | Pathway-based clinical representation |
| --- | --- | --- |
| Stroke: ADL/IADL | MBI, Barthel Index, IADL score | Task-specific compensatory strategy, cognitive-motor coupling, fatigue-related performance variability, environmental participation pattern |
| Stroke: motor recovery | Brunnstrom stage; FMA-UE score | Pathological synergy vs. compensatory recruitment distinction; inter-joint coordination trajectory; trunk compensation ratio; corticospinal reorganization pathway |
| Stroke: upper-limb dexterity | Box and Block Test count | Wrist, shoulder, and trunk compensation quantified by inertial sensors; reach trajectory quality; bilateral coordination asymmetry |
| Balance | BBS total score | Sensory dependence, correction latency, sway dynamics, task-transition vulnerability |
| Mobility | TUG time | Sit-to-stand control, gait initiation, turning instability, recovery after perturbation |
| Cancer survivorship | ICD code for gait abnormality or fall | Treatment-related sensory impairment → instability → fear → activity restriction → disability |
| Symptoms | Symptom severity score | Experienced symptom → somatic sensation → interpretation → function/disability pathway |
| Performance status | ECOG/KPS | Daily activity trajectory, variability, reserve, deterioration, and recovery pattern |
| Breast cancer-related lymphedema | ISL stage; arm circumference | Fluid burden (bioimpedance), lymphatic transport integrity (ICG lymphography), tissue remodeling (ultrasound), symptom/function burden; discordance among domains as a scale problem rather than measurement error |

# Supplementary Box 1. Illustrative CRTC walk-through: compensatory upper-limb motor pattern in a subacute stroke patient

| CRTC stage | Example application |
| --- | --- |
| Clinical situation capture | Video and inertial sensor capture during reaching and Box and Block Test in a patient with left hemiplegia scored at Brunnstrom stage IV |
| Phenomenon selection | Compensation-dominant upper-limb motor pattern rather than global “impaired dexterity” |
| Event segmentation | Reach initiation, grasp, transport, release, and return phases; within-session trial-by-trial variability |
| Pathway modeling | Corticospinal tract disruption → pathological flexor synergy → trunk rotation and shoulder elevation recruited to achieve reach → compensatory pattern consolidation → reduced distal motor recovery |
| Digital representation | Trunk rotation angle, shoulder elevation trajectory, elbow extension range, wrist deviation, inter-joint timing, and compensation ratio per trial |
| Clinical semantic mapping | Compensation-dominant reach strategy with progressive trunk and shoulder overuse |
| Computable phenotype construction | Compensation-dominant upper-limb phenotype |
| AI inference | Probability of true motor restoration vs. compensation-dependent functional plateau; predicted response to constraint-induced or task-specific training |
| Clinical return | Targeted task-specific motor training, constraint-based approaches, real-time feedback on compensation degree, and trajectory monitoring across the rehabilitation course |

# Supplementary Box 2. Illustrative CRTC walk-through: transition instability in a breast cancer survivor after chemotherapy

| CRTC stage | Example application |
| --- | --- |
| Clinical situation capture | Video or wearable capture during chair rise, gait initiation, turning, and early walking in a survivor reporting unsteadiness |
| Phenomenon selection | Instability during transitional movement rather than global “slow mobility” |
| Event segmentation | Sit-to-stand, weight transfer, first step, turn initiation, turn completion, and recovery phase |
| Pathway modeling | Chemotherapy-related sensory disturbance → delayed postural correction → cautious transition → perceived instability → fear of falling → activity restriction |
| Digital representation | Mediolateral sway, correction latency, turning variability, step-timing irregularity, visual dependence, and trunk strategy |
| Clinical semantic mapping | Dynamic balance vulnerability during task transitions |
| Computable phenotype construction | Transition-instability phenotype |
| AI inference | Risk of fall, mobility decline, reduced community participation, or poor response to capacity-focused exercise alone |
| Clinical return | Sensorimotor balance training, transition-specific practice, graded exposure, environmental modification, and patient feedback |
